# A novel remnant liver-first strategy for liver autotransplantation in patients with end-stage hepatic alveolar echinococcosis: a retrospective case series

**DOI:** 10.1097/JS9.0000000000000604

**Published:** 2023-09-02

**Authors:** Tao Lv, Gang Xu, Xi Xu, Gang Wu, Chen-Fei Wan, Jiu-Lin Song, Jian Yang, Yong-Jie Zhou, Kui Luo, Hong Wu, Cheng-Jie Ye, Lv-Nan Yan, Wan-Yee Lau, Jia-Yin Yang

**Affiliations:** a Liver Transplant Center, Organ Transplant Center; b Laboratory of Liver Transplantation, Key Laboratory of Transplant Engineering and Immunology, NHC; c Department of Radiology, Huaxi MR Research Center (HMRRC), National Clinical Research Center for Geriatrics, Frontiers Science Center for Disease-Related Molecular Network, State Key Laboratory of Biotherapy, West China Hospital of Sichuan University, Chengdu; d Department of Hepatobiliary Surgery, Qinghai Provincial People’s Hospital, Xining; e Faculty of Medicine, The Chinese University of Hong Kong, Shatin, Hong Kong, SAR, People’s Republic of China

**Keywords:** case series, end-stage hepatic alveolar echinococcosis, in vivo liver resection, liver autotransplantation, liver transplantation, remnant liver

## Abstract

**Background:**

*Ex vivo* liver resection combined with autotransplantation is an effective therapeutic strategy for unresectable end-stage hepatic alveolar echinococcosis (HAE). However, *ex vivo* liver resection combined with autotransplantation is a technically demanding and time-consuming procedure associated with significant morbidity and mortality. The authors aimed to present our novel remnant liver-first strategy of *in vivo* liver resection combined with autotransplantation (IRAT) technique for treating patients with end-stage HAE.

**Methods:**

This retrospective study included patients who underwent IRAT between January 2014 and December 2020 at two institutions. Patients with end-stage HAE were carefully assessed for IRAT by a multidisciplinary team. The safety, feasibility, and outcomes of this novel technique were analyzed.

**Results:**

IRAT was successfully performed in six patients, with no perioperative deaths. The median operative time was 537.5 min (range, 501.3–580.0), the median anhepatic time was 59.0 min (range, 54.0–65.5), and the median cold ischemia time was 165.0 min (range, 153.8–201.5). The median intraoperative blood loss was 700.0 ml (range, 475.0–950.0). In-hospital complications occurred in two patients. No Clavien–Dindo grade III or higher complications were observed. At a median follow-up of 18.6 months (range, 15.4–76.0) , all patients were alive. No recurrence of HAE was observed.

**Conclusion:**

The remnant liver-first strategy of IRAT is feasible and safe for selected patients with end-stage HAE. The widespread adoption of this novel technique requires further studies to standardize the operative procedure and identify patients who are most likely to benefit from it.

## Introduction

Highlights

- The *ex vivo* liver resection combined with autotransplantation is a technically demanding and time-consuming procedure with significant morbidity and mortality rates.
- We introduce a novel surgical strategy for the management of end-stage hepatic alveolar echinococcosis.
- The novel remnant liver-first strategy facilitates a more flexible and precise revascularization of autografts.
- This time-saving technique ensures sufficient surgical space and better exposure during the operation, which improves the outcomes with less blood loss and shorter anhepatic and cold ischemia time.
- This technique is a simplified, safe, and feasible alternative to traditional procedure.

Alveolar echinococcosis (AE) is a rare but life-threatening parasitic disease that occurs primarily in temperate climate zones in the northern hemisphere, including China, northern Japan, central Asia, the central part of western Europe, the Mediterranean, and Russia^1^. Approximately 18 000 new AE cases are diagnosed annually worldwide, of which 91% occur in western China^2^. Almost all primary AE lesions affect the liver, and the disease can remain asymptomatic for 10–15 years. When symptoms manifest and are left untreated, the mortality rate can be up to 90% within 10 years of diagnosis^3^. Albendazole therapy and surgery are the cornerstones of treatment for hepatic alveolar echinococcosis (HAE)^4^. However, HAE tends to be underdiagnosed, and typically follows an indolent course until symptoms or complications develop. Over the past decades, considerable advances have been made to improve the diagnosis and management of HAE; however, approximately one-third of newly diagnosed cases remain untreatable by conventional radical liver resection^5–7^. Liver transplantation (LTx), specifically *ex vivo* liver resection combined with autotransplantation (ERAT), is a salvage therapy for end-stage HAE when lesions exceed conventional resectability limits^6,8–10^. With increasing progress and experience accumulation, ERAT is no longer an experimental surgery but an established procedure, which plays a clinically important role in selected situations. Recently, our center has reported the experience of employing this surgical technique in their setup^7,9–12^. The results showed that ERAT was significantly beneficial in selective end-stage HAE patients compared with nonsurgical treatment. Compared to conventional LTx, ERAT avoids the use of immunosuppressive drugs, which may increase the risk of local disease recurrence and the development of extrahepatic metastases. However, ERAT is a technically challenging procedure that involves multiple complex surgical steps to maintain hemodynamic stability during the anhepatic phase, including *en bloc* liver resection, portosystemic shunting, and caval reconstruction^8,9^. These steps result in prolonged operative time and increased cold ischemia time (CIT) of the autologous grafts, which increases postoperative morbidity rates^10^. *En bloc* liver resection is particularly challenging in patients with large lesions that have invaded the diaphragm and porta hepatis.

As such, the above-mentioned issues have encouraged the modification of the surgical technique for end-stage HAE. Herein, we introduce a novel remnant liver-first strategy for *in vivo* liver resection combined with autotransplantation (IRAT) in well-selected patients with end-stage HAE and propose some initial indications for this procedure.

## Patients and methods

### Case series

**Figure 1:**
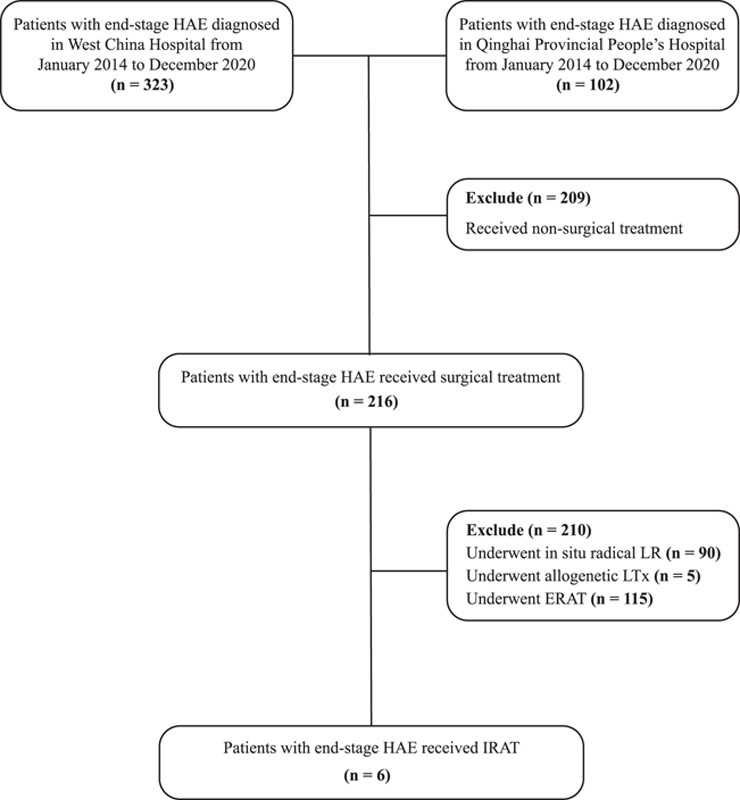
Schematic flow diagram illustrating the selection process for patients with end-stage end-staged hepatic alveolar echinococcosis (HAE) underwent *in vivo* liver resection combined with autotransplantation (IRAT) in the present study. LR, liver resection; LTx, liver transplantation; ERAT, *ex vivo* liver resection combined with autotransplantation.

Between January 2014 and December 2020, 425 patients with end-stage HAE were treated at two tertiary referral centers (Fig. 1). The patients were classified according to the WHO PNM staging system for AE^13^. This study was conducted in accordance with the principles of the Declaration of Helsinki. The study was approved by the Ethics Committees of West China Hospital (NO. 2022-1992) and Qinghai Provincial People’s Hospital (NO. 2022-251) and registered in the Chinese Clinical Trial Registry (ChiCTR2300067330). The requirement for informed patient consent was waived because of the retrospective nature of the study. This study was conducted according to the Preferred Reporting Of Case Series in Surgery (PROCESS) Criteria^14^.

**Figure 2:**
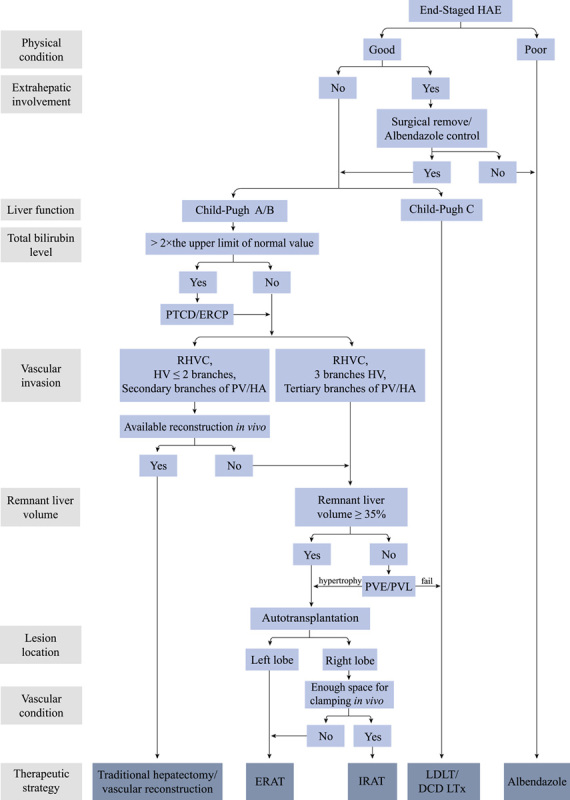
The algorithm of therapeutic strategies for patients with end-staged hepatic alveolar echinococcosis (HAE). PTCD, percutaneous transhepatic cholangial drainage; ERCP, endoscopic retrograde cholangiopancreatography; RHVC, retrohepatic vena cava; HV, hepatic vein; PV, portal vein; HA, hepatic artery; PVE, portal vein embolization; PVL, portal vein ligation; ERAT, *ex vivo* liver resection combined with autotransplantation; IRAT, *in vivo* liver resection combined with autotransplantation; LDLT, living-donor liver transplantation; DCD LTx, donation after circulatory death liver transplantation.

Overlapping surgical teams at these centers perform cadaveric donor LTx, living-donor LTx, and liver autotransplantation. The first orthotopic LTx for end-stage HAE was performed in the early 2000s, and the first ERAT for end-stage HAE was performed in 2014^9,15^. Since then, a multidisciplinary team has been formed that includes hepatobiliary surgeons, vascular surgeons, hepatologists, radiologists, and anesthetists to develop a robust LTx management algorithm for HAE (Fig. 2). Between January 2014 and December 2020, six patients with the following critical clinical features were selected to undergo IRAT: end-stage HAE assessed as unresectable through conventional hepatectomy combined with vascular reconstruction *in vivo*; good physiological state and liver function (Child-Pugh Grade A/B)^16^; involvement of the retrohepatic inferior vena cava (IVC) and more than two hepatic vein branches; invasion of secondary or tertiary branches of the portal vein (PV) and hepatic arteries (HAs); preoperative serum total bilirubin level less than or equal to 2 × the upper limit of normal reference range; hyperplastic remnant liver with preoperative assessment of the remnant liver volume to standard liver volume ratio (RLV/SLV) greater than or equal to 0.35; lesions located in the right liver; and sufficient space to allow placement of a tie or a vascular clamp *in vivo* during the procedure. Patients with extrahepatic lesions that could not be surgically removed and patients treated with albendazole were excluded.

### Preoperative evaluation

**Figure 3:**
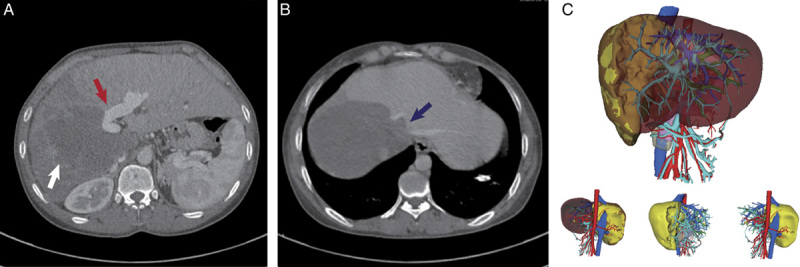
The preoperative imaging assessment and surgical procedures of ‘remnant-liver-first’ strategy for IRAT in patients (case 3) with end-stage HAE. (A) and (B) Preoperative CT demonstrated a giant lesion occupying the entire right liver and left medial section of the liver (white arrow) that invaded portal veins (red arrow) and retrohepatic inferior vena cava (blue arrow); (C) The three-dimensional reconstruction of the lesion, which provided information of remnant liver volume and visualized the vascular and biliary tract anatomy. IRAT, *in vivo* liver resection combined with autotransplantation; HAE, hepatic alveolar echinococcosis; CT, computed tomography.

All six patients enrolled in both institutions were evaluated by blood assays and imaging examination, including computed tomography (Fig. 3A and B) and MRI, to evaluate liver function, hepatic lesions, and vascular involvement, and to detect extrahepatic metastases. Positron emission tomography-computed tomography was performed if extrahepatic metastases were clinically suspected. Three-dimensional imaging analysis software (IQQA-Liver; EDDA Technology, Inc.) was used to calculate the RLV and to visualize the vascular anatomy and spatial locations of the hepatic lesions (Fig. 3C). A RLV/SLV of at least 35% was set as the threshold for safe resection based on our previous reports^10,12^. Patients with preoperative obstructive jaundice and serum total bilirubin levels more than twice the upper normal limit underwent either percutaneous transhepatic cholangial drainage or endoscopic retrograde cholangiopancreatographic drainage to reduce serum bilirubin to normal levels.

### Surgical procedures

#### In vivo liver autograft harvesting based on the remnant liver-first strategy

**Figure 4:**
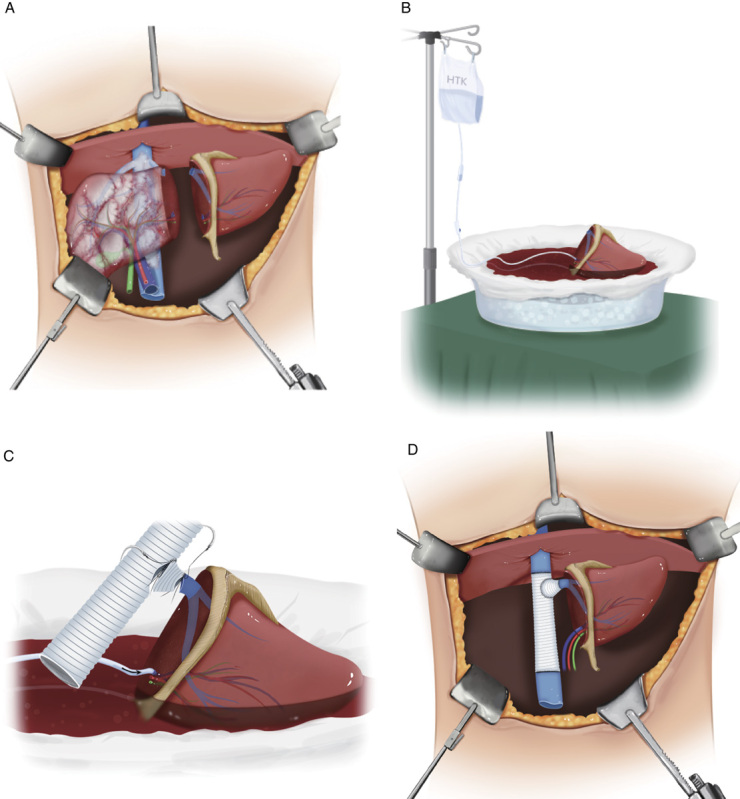
Surgical procedures of ‘remnant-liver-first’ strategy for IRAT in patients with end-stage HAE. (A) The schematic diagram demonstrated the HAE had lesion invaded the entire liver and related vascular and biliary tract except the left lateral section *in vivo* liver resection for left lateral section was carried out and used as autograft; (B) The schematic diagram of hypothermic perfusion for autograft with a HTK solution; (C) The schematic diagram of *ex vivo* back-table revascularization of IVC and LHV (outflow of autograft) through using artificial vascular grafts; (D) The schematic diagram of the vascular and biliary tract reconstruction and state of the liver autograft implanted to the orthotopic position. IRAT, *in vivo* liver resection combined with autotransplantation; HAE, hepatic alveolar echinococcosis; HTK solution, histidine-tryptophan-ketoglutarate solution; IVC, inferior vena cava; LHV, left hepatic vein.

A Mercedes incision was made to ensure adequate exposure of the liver tissue (Fig. 4A). After carefully excluding the presence of any extrahepatic lesions in the abdomen, intraoperative ultrasonography was used to evaluate the presence of intrahepatic microlesions and the planned liver parenchymal transection plane. The hepatocaval confluence and hepatic hilum were carefully examined to ensure that the hepatic vasculature of the left liver had sufficient space for a tie or vascular clamp to pass through. The limits of the liver resection were marked on the liver surface using an electrosurgical cautery device. The proper HA was ligated and divided, followed by the left PV and bile duct. The liver parenchyma was transected with a minimum 1.0 cm margin using a Cavitron ultrasonic surgical aspirator (CUSA, Valleylab), a harmonic scalpel (Ethicon), and Hem-o-Lok clips (Weck, Telefex Medical). Bleeding and bile leakage from cut liver surfaces were carefully controlled. To ensure microscopic margin-negative resection, frozen sections of surgical margins were examined. Other aspects of the liver parenchymal transection were similar to those of allografts harvested from living donors, as reported in our previous studies^17,18^.

#### Ex vivo liver autograft preparation and vascular reconstruction

After successful harvesting of the left lateral liver section, hypothermic perfusion of the autograft was immediately performed using a 0−4°C histidine-tryptophan-ketoglutarate solution (HTK solution; Dr Franz Kohler Chemie) (Fig. 4B). The IVC and left hepatic vein (LHV), which were the outflows of the autograft, were reconstructed using artificial vascular grafts (InterGard, InterVascular SAS, Inc.) (Fig. 4C). Alternatively, autologous vessels, such as the greater saphenous vein, were used for the reconstruction of the IVC and LHV. HA and PV defects were patched and/or bridged using artificial vascular grafts or autologous vessels.

#### In vivo resection of the remaining AE-involved liver

After completion of the liver autograft harvesting and preparation of the autograft by the harvesting team of surgeons, another surgical team simultaneously performed *en bloc in vivo* resection of the remaining AE-involved liver with the retrohepatic vena cava. The remaining AE-involved liver was carefully dissected from the adjacent adhesions. The surgical instruments and procedures were the same as that described above for liver autograft harvesting. Local resection of part of the diaphragm was performed in patients with diaphragmatic involvement. All resected specimens were subjected to histopathological examination.

#### Autotransplantation

Upon completion of the resection of the remaining AE involving the liver, the autograft with the reconstructed vessels was placed in an orthotropic position (Fig. 4D). Reperfusion was initiated after successful reconstruction of the IVC and PV. The anhepatic phase was defined as the time from the removal of the remaining AE-involved liver to reperfusion of the autograft. HA and biliary duct reconstructions were performed. Finally, the autograft was carefully assessed to detect any possible bile leakage and bleeding.

### Postoperative management and follow-up assessment

After surgery, all patients were transferred to the ICU. The length of hospital stay was defined as the number of days from patient admission to hospital discharge. The length of the ICU stay was defined as the number of days the patient stayed in the ICU before being transferred back to the general ward. Standard prophylactic anticoagulants and antiaggregate agents were administered postoperatively to all patients with prosthetic graft replacement to prevent thrombotic complications. Postoperative biliary leakage was defined according to the International Study Group for Liver Surgery criteria^19^. Postoperative complications were graded according to the Clavien–Dindo classification^20^. Mortality was defined as death occurring from the time of surgery until 90 days after autotransplantation. All patients underwent albendazole treatment for two consecutive years following surgery. Follow-up examinations, including liver function, serological, and imaging tests, were performed once every 3–6 months after discharge.

## Results

### Demographic characteristics of enrolled patients

Between January 2014 and December 2020, a total of 203 patients underwent surgical treatment at two institutions, of which, six patients who underwent IRAT was included. Of these patients, four patients underwent IRAT at West China Hospital and two patients underwent IRAT at Qinghai Provincial People's Hospital (Fig. 1). The demographic characteristics for all six patients are summarized in Table 1. The mean age of the patients was 37 years (range, 21–50). All of the patients had end-staged HAE, the median size of these lesions was 15.75 cm (range, 14.8–17.3). The PNM stage of HAE was IV (P4N1M0) in five patients (83.33%) and IIIa (P3N0M0) in one patient (16.67%). All the measured values of RLV/SLV were higher than 35%, and the mean actual RLV/SLV was 60.34% (range 51–77%). The median intraoperative weight of the autograft was 570.0 g (range, 515.0–820.0). Among them, four patients (66.7%) (patients 2, 3, 5, and 6) presented with obstructive jaundice. Preoperative serum total bilirubin levels were more than twice the upper limit of the normal range. Three patients underwent percutaneous transhepatic cholangial drainage and one patient underwent endoscopic retrograde cholangiopancreatographic prior to surgery to reduce bilirubin levels.

**Table 1:** Clinical characteristics of the enrolled patients with end-staged HAE underwent IRAT.

	Patient 1	Patient 2	Patient 3	Patient 4	Patient 5	Patient 6
Age (years)	37	34	43	50	21	37
Sex	Female	Female	Male	Male	Male	Female
BMI (kg/m^2^)	25.6	24.4	23.5	26.7	21.8	20.1
RLV/SLV (%)	850.0/1340.0 (63.0)	630.0/1050.0 (60.0)	620.0/900.0 (77.0)	590.0/1050.0 (56.0)	545.0/990.0 (55.0)	560.0/1100.0 (51.0)
Autograft mass (g)	820.0	600.0	710.0	540.0	515.0	525.0
Lesion size (cm)	17.3	16.5	16.1	15.4	14.8	15.1
PNM stage	P3N0M0	P4N1M0	P4N1M0	P4N1M0	P4N1M0	P4N1M0
MELD score	3	19	12	7	11	14
MELD-Na score	7	22	16	7	11	18
Child-pugh grade	A	B	B	A	A	B
TBIL (umol/l)	5.8	223.0	71.1	15.8	59.8	112.0
ALT (IU/l)	32.0	48.0	29.0	60.0	183.0	53.0
AST (IU/l)	33.0	47.0	36.0	90.0	151.0	39.0
INR	1.1	1.3	1.0	1.1	1.0	1.0
Crea (umol/l)	58.0	48.0	70.0	84.0	47.5	57.0
PTCD/ERCP	–	PTCD	PTCD	–	PTCD	ERCP

ALT, alanine aminotransferase; AST, aspartate transaminase; Crea, creatinine; ERCP, endoscopic retrograde cholangiopancreatography; INR, international normalized ratio; PTCD, percutaneous transhepatic cholangial drainage; RLV, estimated remnant liver volume; SLV, estimated standard liver volume; SLV=706.2×BSA+2.4, BSA=0.007184× Height (cm)^0.725^×Body Weight (kg)^0.425^; TBIL, total bilirubin.

### Surgical parameters of enrolled patients

Table 2 shows surgical and perioperative parameters for enrolled patients from subjects in our study. The median overall operative time was 537.5 min (range, 501.3–580.0 min). The median anhepatic time was 59.0 min (range, 54.0–65.5 min), and the median CIT was 165.0 min (range, 153.8–201.5 min). The median estimated blood loss during the procedure was 700.0 ml (range, 475.0–950.0 ml), and all patients received a blood transfusion. The details pertinent to vascular and biliary reconstructions are presented in Table 2.

**Table 2:** Perioperative parameters and postoperative outcomes of the clinical series.

	Patient 1	Patient 2	Patient 3	Patient 4	Patient 5	Patient 6	Overall value
Perioperative parameters							
Overall operative time (min)	515.0	490.0	505.0	570.0	610.0	560.0	537.5 (501.3–580.0)
Autograft reconstruction time (min)	55.0	30.0	47.0	35.0	40.0	35.0	37.5 (33.8–49.0)
Diseased liver removal time (min)	110.0	105.0	100.0	150.0	130.0	105.0	107.5 (103.8–135.0)
Anhepatic phase (min)	57.0	45.0	60.0	65.0	67.0	58.0	59.0 (54.0–65.5)
Cold ischemia time (min)	167.0	135.0	160.0	215.0	197.0	163.0	165.0 (153.8–201.5)
Reconstruction pattern							
Inferior vena cava	H2	H2	H2	H2	H2	H2	—
Portal vein	P1	P2	P1	P1	P1	P2	—
Hepatic artery	A1	A1	A1	A2	A1	A1	—
Biliary tract	B1	B1	B2	B3	B3	B3	—
EBL (ml)	400.0	500.0	650.0	1100.0	750.0	900.0	700.0 (475.0–950)
Blood transfusion							
Suspended RBC (U)	4.0	6.0	4.0	6.0	5.0	8.0	5.0 (4.0–6.5)
Plasma (ml)	0.0	400.0	400.0	500.0	400.0	600.0	400.0 (300.0–525.0)
Postoperative outcomes							
Postoperative ICU LOS (days)	3.0	2.0	2.0	4.0	2.0	1.0	2.0 (1.8–3.3)
Postoperative hospital LOS (days)	10.0	9.0	10.0	13.0	18.0	12.0	11.0 (9.8–14.3)
Postoperative complications	None	None	None	Pneumonia	None	Biliary leakage	—
Clavien–Dindo Classification	—	—	—	II	—	II	—
Follow-up (months)	12.5	16.3	18.5	18.7	76.0	76.0	18.6 (15.4–76.0)
Current status	Alive	Alive	Alive	Alive	Alive	Alive	—

NOTE: Data of overall value are provided as median (IQR).

EBL, estimate blood loss; IVC, Inferior vena cava; LOS, length of stay; RBC, red blood cell.

H1: hepatic vein or extended hepatic vein with segmental grafts or repaired with patch grafts to IVC (end to end); H2: hepatic vein or extended hepatic vein with segmental grafts or repaired with patch grafts to prosthetic graft (end to side); P1: autograft portal vein to portal trunk (end to end); P2: sagittal portion of left portal vein to portal trunk (end to end); A1: autograft hepatic artery to the proper hepatic artery; A2: bridged hepatic artery with autologous vessel to the proper hepatic artery; B1: autograft biliary duct to common bile duct; B2: sagittal portion of left hepatic duct to common bile duct; B3: Choledechojejunostomy.

### Postoperative complications, follow-up, and long-term outcomes

**Figure 5:**
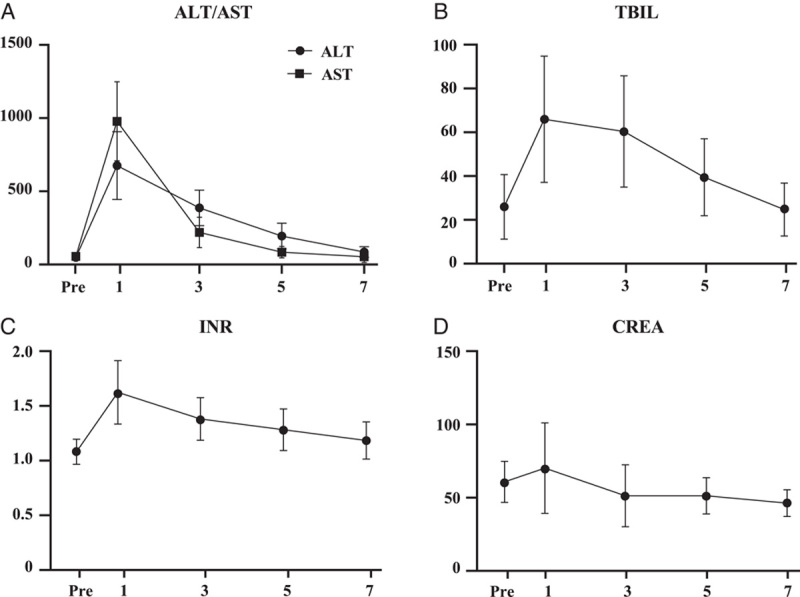
The postoperative results of laboratory tests. (A), (B), and (C) The changes of liver function (ALT, AST, TBIL, INR) of patients after surgery; (D) The changes of renal function (CREA) of patients after surgery. ALT, alanine aminotransferase; AST, aspartate aminotransferase; INR, international normalized ratio; TBIL, total bilirubin; CREA, creatinine.

**Figure 6:**
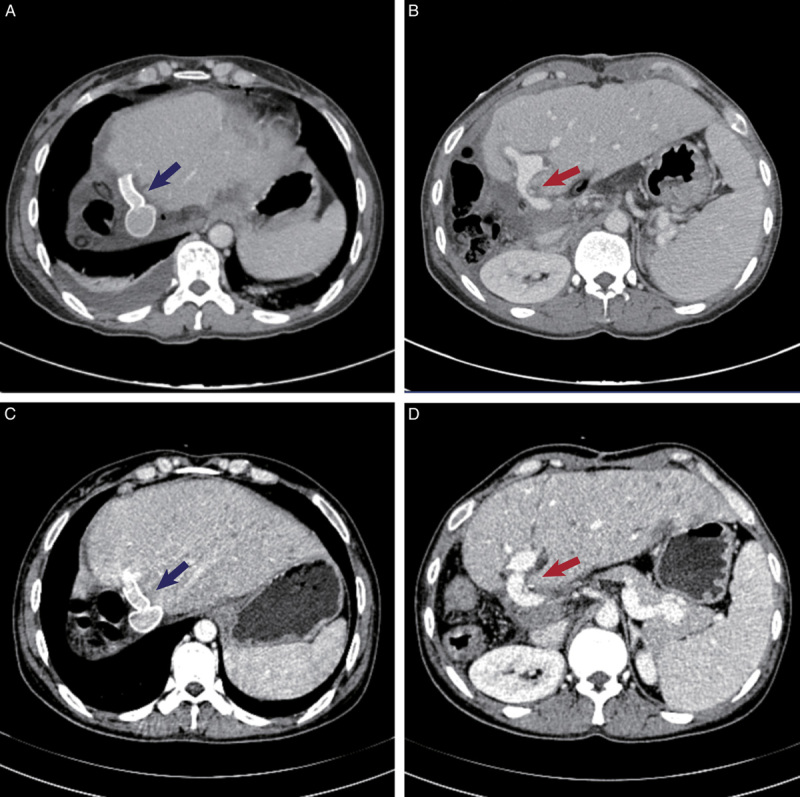
The postoperative results of CT scan. (A), (B), (C), and (D) The CT scan of Patient 3, 1 month and 1 year after surgery, respectively. As demonstrated, regenerated liver with patent reconstructed vessels and no HAE recurrence. The inferior vena cava and left hepatic vein reconstructed by artificial vascular grafts (blue arrow), portal veins (red arrow). CT, computed tomography.

Postoperative laboratory tests of patients are shown in Figure 5. The perioperative mortality was zero. As shown in Table 2, postoperative complications occurred in two patients (33.3%); however, none of the patients had Clavien–Dindo grade III or higher postoperative complications. Biliary leakage occurred in one patient (patient 6) and was conservatively treated with an extended drainage duration. One patient (patient 4) developed pneumonia, which was successfully treated with antibiotic therapy. After a median follow-up of 18.6 months (range, 15.4–76.0), all patients were alive and reported normal activities of daily living. None of the patients developed AE recurrences or extrahepatic metastases. A representative case (patient 3) of the imaging result are presented in Figure 6.

## Discussion

HAE behaves as a slow-growing liver cancer with progressive local invasion involving the biliary and vascular systems and surrounding organs. Most patients with HAE are diagnosed at an advanced stage of the disease losing the opportunity to undergo conventional radical liver resection. Since 1988, when Pichlmayr *et al*.^21^ first reported ERAT as the treatment for a patient with liver metastases of a leiomyosarcoma, this surgical technique has been described in many patients with various types of unreachable advanced hepatic malignant tumors, as well as nonmalignant lesions^22^. In recent years, ERAT has been widely used for the treatment of end-stage HAE and has shown promising outcomes^6,9,10,23^. To date, our center has performed more than 100 ERAT operations and has developed considerable expertise in this field. However, this treatment remains technically challenging and the long-term stability outcomes remain controversial. A published study enrolled two European cohort (involving 111 patients in Bern, Switzerland, and 172 patients in Besançon, France, respectively) showed that albendazole alone or in conjunction with endoscopic biliary stenting was an acceptable treatment for patients with end-stage HAE^24^. However, compared to our patients, the patients enrolled in these two cohorts were older and were diagnosed with HAE at an earlier stage^6,10,24^. It has been noted that the strict follow-up required is likely to be more challenging for patients living in remote regions such as those located far from the referral centers in China^24,25^. Furthermore, Schmidberger *et al*.^26^ recently compared the quality of life of patients who underwent curative resectional surgery with that of patients who underwent conservative medical treatment, suggesting that medical treatment was not inferior to surgery in terms of mental and physical quality of life. However, LTx may be a better definitive treatment modality in patients with unresectable lesions. Recently, a study on the largest cohort of patients with end-stage HAE reported from our center analyzing the benefits and risks of ERAT showed that ERAT significantly improved the long-term survival of patients with end-stage HAE when compared with nonsurgical treatment (5-year survival, 82.1 vs. 19.1%, *P*<0.05)^10^. It follows that ERAT is clearly a good choice for patients with end-stage HAE.

However, in addition to the technical challenges and longer operation time of ERAT, both the anhepatic phase and the CIT are relatively long^6,9^. With large-sized HAE and severe infiltration of vascular or biliary structures, *en bloc* resection of the entire liver can increase the risk of intraoperative bleeding. As both blood vessels and bile ducts are not easily assessed *in vitro*, there may be an increased risk of perioperative complications such as biliary leakage, hemorrhage, and liver failure (Table 3). These factors motivated us to explore an alternative, modified surgical approach. The proposed IRAT, to some extent, overcomes the aforementioned drawbacks of ERAT, which may further reduce surgical complications and the perioperative mortality rate^22^. In the present study, six patients underwent radical treatment for end-stage HAE with the remnant liver-first strategy for liver autotransplantation and exhibited good outcomes. The incidence of complications and perioperative mortality rate were lower than the reported surgical outcomes of EART (Table 3). Certainly, whether IRAT has significant advantages in reducing the risk of complications (e.g. biliary leakage, liver failure, bleeding, pleural effusion, renal insufficiency, wound infection, and sepsis) warrants further investigations on a larger population will be required.

**Table 3:** Perioperative details of ERAT for liver diseases from published studies (≥3 cases).

Study	Year	*N*	Diagnosis	Operative time (h)	Anhepatic time (min)	CIT (min)	EBL (ml)	Morbidity/mortality rate
Cancerous tumors (primary hepatic or biliary cancers, metastatic tumors, or other lesions infiltrating on the liver)								
Pichlmayr *et al*.^27^	1990	8	4×CCA, 2×CRCLM, 1×*Met.* LMS, 1×CRC	13.7±2.7	NR	NR	3120.0	62.5% / 37.5%
Lodge *et al*.^28^	2000	4	4×CRCLM	NR	222.5±39.5	NR	NR	75.0% / 25.0%
Oldhafer *et al*.^29^	2000	20	3×HCC, 5×CCA, 3×*Met.* LMS, 9×CRCLM	13.5±2.7	338.2±85.9	NR	NR	NR / 45.0%
Zhang *et al*.^30^	2012	3	2×CCA	6.8±0.5	204.0±50.9	NR	2100.0±953.9	66.7% / 33.3%
Wen *et al*.^31^	2013	3	3×HCC	NR	161.3±41.0	NR	4600.0±2816.0	100.0% / 0.0
Yoon *et al*.^32^	2019	3	1×HCC, 1×CCA, 1×Sarcoma	16.3±1.5	NR	NR	NR	33.0% / 0.0
Noncancerous tumors (HAE)								
Wang *et al*.^33^	2012	6	6×HAE	16.0	NR	300	NR	33.3% / 16.7%
Wen *et al*.^8^	2016	15	15×HAE	15.4±3.1	280.3±67.5	NR	NR	66.7% / 6.6%
Aji *et al*.^6^	2018	69	69×HAE	15.9 (8.0–24.0)	360.0 (104.0-879.0)	NR	1000.0 (400.0–15000.0)	50.7% / 11.5%
Yang *et al*.^9^	2018	31	31×HAE	12.5 (9.4–19.5)	309.00 (180.0-460.0)	NR	1800.0 (1200.0–6000.0)	41.9% / 6.5%
Qiu *et al*.^10^	2022	100	100×HAE	12.4±2.3	310.7±78.7	NR	2300.0 (1600.0–3050.0)	37.0% / 11.0%

CCA, cholangiocarcinoma; CIT, cold ischemia time; CRC, colorectal cancer; CRCLM, colorectal cancer with liver metastases; EBL, estimate blood loss; HAE, hepatic alveolar echinococcosis; HCC, hepatocellular carcinoma; LMS, leiomyosarcoma; *Met*, metastatic; NR, not reported.

Apart from the complex procedure, the limited operative spaces also lead to the time-consuming nature of EART. A published review addressed that HAE lesions are most commonly located on the right side of the liver^25^. This has been attributed to the large angle between the portal trunk and the sagittal branch of the left PV, which might limit parasites from entering the left lateral liver section^6,8–10,25^. Moreover, chronic PV obstruction of the involved right liver and an immune response to the parasite could account for the hypertrophy of the contralateral hepatic parenchyma^2,6^. In the present study, the HAE lesions of the six patients enrolled in our study were all located in the right lobe of the liver. Moreover, the lesions involved the retrohepatic IVC, three hepatic veins, and the hepatocaval confluence. These lesions also involved the tertiary portal and arterial branches, requiring extensive vascular reconstruction. Owing to adjacent adhesions and severe hypertrophy of the contralateral hepatic parenchyma, the operating space was limited. Indeed, all these factors can contribute not only to prolonged operation time, but also to uncontrolled hemorrhage. In our study, to obtain adequate surgical resection margins, the hyperplastic left lateral liver section, which served as the autograft, was removed *in vivo* to facilitate subsequent mobilization of the diseased right liver increasing the operating space and facilitating exposure of the IVC. Furthermore, this approach shortens the anhepatic phase and avoids the need for a temporary portal caval shunt. Additionally, *in vivo* liver resection and autologous LTx enabled one team to resect the diseased liver, whereas another team prepared the remnant liver for bench surgery. The simultaneous procedure by the two teams further shortened the CIT of the autologous graft and the overall operation time. Compared to living-donor LTx, discriminating bile ducts from other vessels is difficult in ERAT as the liver is perfused and bloodless^7,34^. However, blood vessels and bile ducts of the liver can be precisely ligated during *in vivo* liver resection. This may further reduce the risk of reperfused vessel hemorrhage and postoperative biliary leakage. In the present case series, all the enrolled patients required IVC and LHV reconstruction during bench surgery. The *ex vivo* back-table revascularization of autografts can make surgical procedure flexible and precise with no time constraints. In addition, one of the benefits of IRAT over ERAT is that there is no need for temporary caval reconstruction and portosystemic shunting in patients with hepatic veins that are not completely blocked, and this may further shorten the operation time. Furthermore, hypothermic perfusion was performed only for autologous grafts in this study. Considering that parasites grow toward the portal trunk^8^, restricting perfusion solely to the autologous graft may reduce the likelihood of dissemination of the infestation and minimize the postoperative recurrence rate. This hypothesis requires further validation through experimental investigation and a large clinical cohort study.

The removal of the planned autograft is the first step in intestinal autotransplantation. Previous studies have reported that intestinal autograft removal before mesenteric root tumor resection is a feasible operative strategy for patients with intra-abdominal tumors that are considered unresectable using conventional techniques^35,36^. Similar to our findings, although the number of patients who can benefit is small, these remnant autograft-first procedures allow patients with locally advanced lesions to safely undergo surgical treatment. Apart from end-staged HAE, IRAT may be also appropriate for various liver tumors with large size and/or difficult anatomic locations, including a severe compression against or infiltration on large vascular or biliary structures (such as retrohepatic IVC, portal structures, hepatic veins, and hepatocaval confluence).

Our initial experience with six patients indicated that the novel remnant liver-first strategy of IRAT is feasible and safe for treating end-stage HAE. However, our study has several limitations. First, the small sample size and absence of a comparative control group compromised the quality of the study. Second, the follow-up period was short, and only the short-term effects of the IRAT were studied. Nonetheless, IRAT provides a novel radical treatment option for end-stage HAE with distinct therapeutic advantages. Further follow-up and well-designed prospective studies with large sample sizes are required to identify the indications and determine the long-term outcomes of this technique.

## Conclusion

In conclusion, experienced transplant centers, particularly those performing high-volume LTx, can reasonably implement this novel remnant liver-first strategy for liver autotransplantation in carefully selected patients. Since the skillset necessary for IRAT overlaps with living-donor LTx, we believe that these novel surgical strategy will be widely accepted by surgeons in the future.

## Ethical approval

The study was conducted in accordance with the Declaration of Helsinki. The study was approved by the Ethics Committee of West China Hospital (No. 2022-1992) and Qinghai Provincial People’s Hospital (No .2022-251) and registered with the Chinese Clinical Trial Registry (ChiCTR2300067330).

## Consent

Written informed consent was obtained from the patient for publication and any accompanying images. A copy of the written consent is available for review by the Editor-in-Chief of this journal on request.

## Sources of funding

This study was funded by the National Natural Science Foundation of China (No. 82070674), the Natural Science Foundation of Sichuan Province (No. 2022NSFSC0843), the Sichuan Science and Technology Program (No. 2019YFG0036), the China Postdoctoral Science Foundation (No. 2022M712262), the Sichuan Province Key Research and Development Project (No.2021YFS0098), and the Second Batch of High-Level Health Talent Introduction Plan of Qinghai Province.

## Author contribution

J.-Y.Y., C.-J.Y., T.L., G.X., and X.X.: conception of the work; T.L., G.X., G.W., C.-F.W., J.-L.S., H.W., and J.Y.: data collections; T.L., X.X., and Y.-J.Z.: analyzed the data; T.L., G.X., X.X., W.-Y.L., and J.-Y.Y.: wrote the paper; L.-N.Y., L.K., T.L., G.X., X.X., W.-Y.L., and J.-Y.Y.: revised the paper. All authors contributed in final approval of manuscript .

## Conflicts of interest disclosure

The authors declare that they have no conflicts of interest.

## Research registration unique identifying number (UIN)

1. Name of the registry: Chinese Clinical Trial Registry chictr.org.cn.
2. Unique identifying number or registration ID: ChiCTR2300067330.
3. Hyperlink to your specific registration (must be publicly accessible and will be checked): https://www.chictr.org.cn/showproj.aspx?proj=188692.

## Guarantor

Wan-Yee LAU and Jia-Yin Yang.

## References

[1] McManus DP Zhang W Li J. Echinococcosis. Lancet 2003;362:1295–1304. 14575976 10.1016/S0140-6736(03)14573-4

[2] Peters L Burkert S Grüner B. Parasites of the liver - epidemiology, diagnosis and clinical management in the European context. J Hepatol 2021;75:202–218. 33636243 10.1016/j.jhep.2021.02.015

[3] Kern P. Clinical features and treatment of alveolar echinococcosis. Curr Opin Infect Dis 2010;23:505–512. 20683265 10.1097/QCO.0b013e32833d7516

[4] McManus DP Gray DJ Zhang W. Diagnosis, treatment, and management of echinococcosis. BMJ 2012;344:e3866. 22689886 10.1136/bmj.e3866

[5] Grüner B Kern P Mayer B. Comprehensive diagnosis and treatment of alveolar echinococcosis: a single-center, long-term observational study of 312 patients in Germany. GMS Infect Dis 2017;5:Doc01. 30671323 10.3205/id000027 PMC6301735

[6] Aji T Dong JH Shao YM. Ex vivo liver resection and autotransplantation as alternative to allotransplantation for end-stage hepatic alveolar echinococcosis. J Hepatol 2018;69:1037–1046. 30031886 10.1016/j.jhep.2018.07.006

[7] Yang C He J Yang X. Surgical approaches for definitive treatment of hepatic alveolar echinococcosis: results of a survey in 178 patients. Parasitology 2019;146:1414–1420. 31267889 10.1017/S0031182019000891

[8] Wen H Dong JH Zhang JH. Ex vivo liver resection and autotransplantation for end-stage alveolar echinococcosis: a case series. Am J Transplant 2016;16:615–624. 26460900 10.1111/ajt.13465

[9] Yang X Qiu Y Huang B. Novel techniques and preliminary results of ex vivo liver resection and autotransplantation for end-stage hepatic alveolar echinococcosis: a study of 31 cases. Am J Transplant 2018;18:1668–1679. 29232038 10.1111/ajt.14621 PMC6055796

[10] Qiu Y Huang B Yang X. Evaluating the benefits and risks of ex vivo liver resection and autotransplantation in treating hepatic end-stage alveolar echinococcosis. Clin Infect Dis 2022;75:1289–1296. 35271705 10.1093/cid/ciac195

[11] Qiu Y Yang X Shen S. Vascular infiltration-based surgical planning in treating end-stage hepatic alveolar echinococcosis with ex vivo liver resection and autotransplantation. Surgery 2019;165:889–896. 30591376 10.1016/j.surg.2018.11.007

[12] Shen S Qiu Y Yang X. Remnant liver-to-standard liver volume ratio below 40% is safe in ex vivo liver resection and autotransplantation. J Gastrointest Surg 2019;23:1964–1972. 30374819 10.1007/s11605-018-4022-4

[13] Kern P Wen H Sato N. WHO classification of alveolar echinococcosis: principles and application. Parasitol Int 2006;55 Suppl:S283–S287. 16343985 10.1016/j.parint.2005.11.041

[14] Agha RA Sohrabi C Mathew G. The PROCESS 2020 guideline: updating Consensus Preferred Reporting Of CasESeries in Surgery (PROCESS) guidelines. Int J Surg 2020;84:231–235. 33189880 10.1016/j.ijsu.2020.11.005

[15] Li F Yang M Li B. Initial clinical results of orthotopic liver transplantation for hepatic alveolar echinococcosis. Liver Transpl 2007;13:924–926. 17538987 10.1002/lt.21187

[16] Pugh RN Murray-Lyon IM Dawson JL. Transection of the oesophagus for bleeding oesophageal varices. Br J Surg 1973;60:646–649. 4541913 10.1002/bjs.1800600817

[17] Wen TF Chen ZY Yan LN. Measures for increasing the safety of donors in living donor liver transplantation using right lobe grafts. Hepatobiliary Pancreat Dis Int 2007;6:590–595. 18086623

[18] Feng X Yuan D Wei YG. Outcomes of adult-to-adult living donor liver transplantation: a single center experience. Chin Med J (Engl) 2009;122:781–786. 19493389

[19] Koch M Garden OJ Padbury R. Bile leakage after hepatobiliary and pancreatic surgery: a definition and grading of severity by the International Study Group of Liver Surgery. Surgery 2011;149:680–688. 21316725 10.1016/j.surg.2010.12.002

[20] Dindo D Demartines N Clavien PA. Classification of surgical complications: a new proposal with evaluation in a cohort of 6336 patients and results of a survey. Ann Surg 2004;240:205–213. 15273542 10.1097/01.sla.0000133083.54934.ae PMC1360123

[21] Pichlmayr R Bretschneider HJ Kirchner E. Ex situ operation on the liver. A new possibility in liver surgery. Langenbecks Arch Chir 1988;373:122–126. 3287072 10.1007/BF01262775

[22] Serrablo A Giménez-Maurel T Utrilla Fornals A. Current indications of ex-situ liver resection: a systematic review. Surgery 2022;172:933–942. 35791978 10.1016/j.surg.2022.04.002

[23] Aliakbarian M Tohidinezhad F Eslami S. Liver transplantation for hepatic alveolar echinococcosis: literature review and three new cases. Infect Dis (Lond) 2018;50:452–459. 29363377 10.1080/23744235.2018.1428823

[24] Beldi G Vuitton D Lachenmayer A. Is ex vivo liver resection and autotransplantation a valid alternative treatment for end-stage hepatic alveolar echinococcosis in Europe? J Hepatol 2019;70:1030–1031. 30718093 10.1016/j.jhep.2018.12.011

[25] Wen H Vuitton L Tuxun T. Echinococcosis: advances in the 21st century. Clin Microbiol Rev 2019;32:e00075–18. 30760475 10.1128/CMR.00075-18 PMC6431127

[26] Schmidberger J Steinbach J Schlingeloff P. Surgery versus conservative drug therapy in alveolar echinococcosis patients in Germany – a health-related quality of life comparison. Food Waterborne Parasitol 2019;16:e00057. 32095627 10.1016/j.fawpar.2019.e00057 PMC7034038

[27] Pichlmayr R Grosse H Hauss J. Technique and preliminary results of extracorporeal liver surgery (bench procedure) and of surgery on the in situ perfused liver. Br J Surg 1990;77:21–26. 2302506 10.1002/bjs.1800770107

[28] Lodge JP Ammori BJ Prasad KR. Ex vivo and in situ resection of inferior vena cava with hepatectomy for colorectal metastases. Ann Surg 2000;231:471–479. 10749606 10.1097/00000658-200004000-00004 PMC1421021

[29] Oldhafer KJ Lang H Schlitt HJ. Long-term experience after ex situ liver surgery. Surgery 2000;127:520–527. 10819060 10.1067/msy.2000.105500

[30] Zhang KM Hu XW Dong JH. Ex-situ liver surgery without veno-venous bypass. World J Gastroenterol 2012;18:7290–7295. 23326135 10.3748/wjg.v18.i48.7290 PMC3544032

[31] Wen PH Lin KH Chen YL. Extracorporeal hepatic resection and autotransplantation using temporary portocaval shunt provides an improved solution for conventionally unresectable HCC. Dig Dis Sci 2013;58:3637–3640. 23918151 10.1007/s10620-013-2801-z PMC3838586

[32] Yoon YI Lee SG Moon DB. Hypothermic perfusion hepatectomy for unresectable liver cancer: a single-center experience. J Hepatobiliary Pancreat Sci 2020;27:254–264. 31562749 10.1002/jhbp.681

[33] Wang H Liu Q Wang Z. Clinical outcomes of Ex Vivo liver resection and liver autotransplantation for hepatic alveolar echinococcosis. J Huazhong Univ Sci Technolog Med Sci 2012;32:598–600. 22886977 10.1007/s11596-012-1003-9

[34] Zeng X Yang X Yang P. Individualized biliary reconstruction techniques in autotransplantation for end-stage hepatic alveolar echinococcosis. HPB (Oxford) 2020;22:578–587. 31471064 10.1016/j.hpb.2019.08.003

[35] Tzvetanov IG Bhati CS Jeon H. Segmental intestinal autotransplantation after extensive enterectomy for removal of large intra-abdominal desmoid tumors of the mesentery root: initial experience. Surgery 2012;151:621–624. 21982069 10.1016/j.surg.2011.07.028

[36] Wu G Zhao Q Wang W. Clinical and nutritional outcomes after intestinal autotransplantation. Surgery 2016;159:1668–1676. 26936523 10.1016/j.surg.2016.01.016

